# A Newly Developed Hematuria Grading System May Predict the Status of Stone-Free and Acute Pyelonephritis of Minimally Invasive Renal Stone Surgery

**DOI:** 10.3390/jcm12082820

**Published:** 2023-04-12

**Authors:** Gyeong Hun Kim, Gyoohwan Jung, Jungyo Suh, Juhyun Park, Sung Yong Cho

**Affiliations:** 1 Department of Urology, Seoul National University Hospital, 101 Daehak-ro, Jongno-gu, Seoul 03080, Republic of Korea; 2 Department of Urology, Hanyang University Seoul Hospital, 222-1, Wangsimni-ro, Seongdong-gu, Seoul 04763, Republic of Korea; 3 Department of Urology, Asan Medical Center, University of Ulsan College of Medicine, 88 Olympic-ro 43-gil, Songpa-gu, Seoul 05505, Republic of Korea; 4 Department of Urology, Seoul National University Hospital, Seoul National University College of Medicine, 101 Daehak-ro, Jongno-gu, Seoul 03080, Republic of Korea

**Keywords:** hematuria grading system, nephrolithiasis, retrograde intra-renal surgery, stone number, stone size

## Abstract

Objectives: The aim of this study was to evaluate the level of hematuria and the presence of clots during retrograde intrarenal surgery (RIRS) and miniaturized percutaneous nephrolithotomy (mPCNL) to predict surgical outcomes. Materials and Methods: The data of patients who underwent RIRS and mPCNL were analyzed separately. A hematuria grading (HG) system was classified into five grades based on the presence of blood clots and any visible stones according to the irrigation settings. Inter-observer reliability of the grading system was assessed using intra-class correlation and Spearman’s rho. Results: The HG system showed high agreement among examiners, with high intra-class reliability and a strong correlation between RIRS and mPCNL groups. The stone density of the Houns-field unit was the most important factor in determining the hematuria across the development and validation groups of RIRS and mPCNL patients. Multivariate logistic regression analysis showed that the HG system was a significant predictor for remnant stones in the PCNL group and the probability of acute pyelonephritis or sepsis in the RIRS group. The high hematuria group showed lower basketing difficulty with the basket with a blue marker instrument than with others. Conclusions: The new HG system shows excellent inter-observer reliability and a correlation with a gradual increase in stone density and surgical difficulty.

## 1. Introduction

Nephrolithiasis is a highly prevalent disease worldwide, ranging from 1–5% in Asia, 5–9% in Europe, and 7–13% in North America [1]. An increasing trend in its prevalence during the last several decades has been reported in developed and developing countries [2,3,4]. Nephrolithiasis is associated with a high lifetime recurrence rate of 50% in the five–ten-year follow-up period and 75% in the 20-year follow-up period [5]. Symptomatic renal stone episodes affect the patient’s quality of life, with a likelihood of renal damage, necessitating the development of an adequate management strategy.

Recently, retrograde intrarenal surgery (RIRS) and miniaturized percutaneous nephrolithotomy (mPCNL) have emerged as safe therapeutic options for managing renal stones, because they can reduce hospitalization time with an acceptable stone-free rate [6,7]. Moreover, the European Association of Urology (EAU) guidelines recommend endoscopic management to treat small-sized stones or stones >2 cm if technically feasible [8]. With the recent development of medical devices and surgical techniques, using RIRS and mPCNL to manage nephrolithiasis is increasing in real-world clinical practice [9].

Because of the small caliber of scope and the nature of underwater surgery, hematuria, especially the presence of blood clots during RIRS and mPCNL, makes surgery more difficult. Several factors, including increased intrarenal pressure, distended cavities in the renal pelvis and renal calyces, urothelial injury by direct laser firing, fragmented stones, and endoscopy manipulations, can cause bleeding during procedures. In addition, the high grade of hematuria can lead to prolonged operating time and affect the stone-free rate. Therefore, it may require an additional operation. However, no studies are available on the correlation of the severity level of hematuria with surgical difficulty or surgical outcomes during these operations.

Therefore, this study aimed to develop and externally validate an easy and reliable hematuria grade (HG) system to assess the severity of hematuria during RIRS and mPCNL. Because the two surgical methods share the fact that hematuria is a key factor for performing surgery and predicting surgical outcomes, our HG system applied the same HG system to both RIRS and mPCNL. We evaluated the intra-class correlation and the inter-observer reliability of the HG system among examiners and determined between-group differences in surgical outcomes based on clinical parameters.

## 2. Methods

### 2.1. Patient Selection and Data Extraction

We retrospectively evaluated the medical records of all patients who underwent RIRS and mPCNL at two institutions. The inclusion criteria followed the guidelines of the Urological Association of Asia [10]. We included all patients with HGs written in operation records. We excluded patients who underwent combined percutaneous nephrolithotomy or simple retrograde ureteroscopy inspection due to a passed stone or suspicion of any abnormality in the upper urinary tract. Patients with preoperative febrile urinary tract infections, a tendency to bleeding, or ureteral strictures were excluded.

This study was approved by the Institutional Review Boards (IRB) of Institution 1 (Approval No.: H-1902-054-1010) and Institution 2 (Approval No.: 10-2019-35). Participants’ waiver of the informed consent requirement was approved by each IRB, considering the retrospective study design involving anonymized data. All experiments were performed following relevant guidelines and regulations.

### 2.2. RIRS Procedures

Three experienced surgeons (S.Y. Cho, J. Park, and J. Suh) at two medical centers performed all procedures with the same surgical method [11,12]. All the patients were placed in the dorsal lithotomy position under general anesthesia, and prophylactic second-generation cephalosporin antibiotics were generally administered 1 h before surgery. A hydrophilic guidewire (Cook Medical Roadrunner^®^ PC Hydrophilic Guide Wire, Terumo Guide Wire Radifocus) was inserted into the ureteral orifice, and retrograde pyelography was performed using a dual-lumen catheter to place a Superstiff guidewire (Boston Scientific Corporation, Marlborough, MA, USA). If a pre-operative JJ stent was inserted, we retracted and put the Superstiff guidewire into the ureter through the JJ stent. The authors placed the Superstiff guidewire very carefully into the kidney so as not to cause damage, and the tip of the guidewire is usually located in the upper major calyx. Subsequently, an 11/13-Fr ureteral access sheath (UAS) (Boston Scientific Corporation) was placed up to the ureteropelvic junction, and a flexible ureteroscope (Flex-X2S and Flex-Xc (Karl Storz, Tuttlingen, Germany) and URF-P5 or URF-V (Olympus, Tokyo, Japan) was inserted through the UAS. The use of a UAS may cause a ureter injury to produce hematuria during RIRS, but when we started the RIRS procedure, we saw no hematuria in all cases. After systemic inspection of the renal calyx, we performed Holmium laser lithotripsy with a Lumenis Pulse™ 100 H or 120 H using a 200 nm laser fiber. In the case of a renal stone lodged in the lower calyx, we retrieved the stone from the upper or middle calyx using a stone basket if feasible. Laser power was set to 1.0 to 2.0 W with a 10–30 Hz frequency. Portions of the fragmented stones were removed with a stone basket, and the type of stone basket varied according to the surgeon’s preferences (Zero Tip Nitinol Stone Retrieval Basket Boston Scientific Corporation, San Jose, CA, USA; SkyLite 1.9 Tipless Nitinol Basket with a blue marker instrument, BD Biosciences, San Jose, CA, USA; NCircle Nitinol Tipless Stone Extractor, Cook Medical, Bloomington, IN, USA). Remnant stones were completely fragmented (stone size < 1 mm) by the pop-dusting procedure. Following laser lithotripsy, we examined the renal calyx for the presence of stones missed during the procedure. Consistent irrigation flow is necessary for endoscopic stone surgery in order to optimize the endoscopic view. An automated irrigation pump, the FloSteady Irrigation Pump (Stryker, Kalamazoo, MI, USA), was used for more consistent flow than irrigation devices that depend on pressure that are manipulated manually with pressure infusion sleeves [13,14]. The maximal pressure setting was 80 mmHg with 1–1.5 L/min for RIRS. To delicately control pressure, a 3-way stop-cock was connected to the irrigation line. Any ureter injuries were documented during the removal of the UAS. Subsequently, a 6-Fr JJ stent was inserted, and a urethral Foley catheter was placed into the bladder. The JJ stent was removed after one to two weeks post-operation.

### 2.3. mPCNL Procedures

As RIRS, mPCNL also was conducted in the same way by all surgeons in this study. Patients were positioned in the Barts “flank-free” modified supine position under general anesthesia [15]. With the aid of a cystoscope, we placed a 5-Fr open-ended ureteral catheter (Cook Medical, Bloomington, IN, USA) into the renal pelvis and used contrast dye to examine the calyceal system. A 12-Fr urethral Foley catheter, which was attached to the ureteral catheter, was used to drain the bladder. Under the assistance of ultrasound and fluoroscopy, an 18-G echo-tip Chiba needle was typically introduced into the mid-pole or lower-pole calyx. A 0.035-inch stiff type ZIPwire™ (Boston Scientific, Marlborough, MA, USA) was then inserted into the renal collecting system or down the ureter through the needle sheath under fluoroscopic guidance. A fascia dilator was used to gradually widen the tract after the skin and fascia were incised. Then, a matched metallic sheath was inserted. A size 12-Fr nephroscope (Karl Storz, Tuttlingen, Germany) was inserted through a 15/16.5-Fr metallic sheath. At the end of the procedure, we used a hemostatic agent without a nephrostomy tube. We used the same automated irrigation pump in mPCNL, the FloSteady Irrigation Pump (Stryker, Kalamazoo, MI, USA), for stable flow. The maximal pressure setting was 50 mmHg 1.0 L/min for the mPCNL.

### 2.4. Establishment and Assessment of the HG System

We developed a five-point HG system (grades 0 to 4) based on the presence of clots, any visible stones, and characteristics of hematuria during RIRS and mPCNL with data obtained from Institution 1 (Table 1). We categorized the severity of hematuria as HG-zero, defined as no hematuria or clot with a clearly visible stone; HG-one, defined as mild hematuria with no clot and a clearly visible stone; HG-two, defined as a visible and unclear stone with scattered hematuria without blood clot; HG-three, defined as hematuria with clots and a clearly visible stone during stone fragmentation; or finally, HG-four, defined as severe hematuria with blood clots posing difficulty in proceeding with surgery. Nurses, first assistants, and certified urologists recorded these HGs at two points (the middle and the end of the operation). Each examiner’s mean value of HGs was rounded to one decimal place.

### 2.5. Development and External Validation Group of the HG System

We analyzed the inter-observer reliability for the HG system among examiners based on intra-class correlation and Spearman’s rho. Reliability was considered suitable when intra-class correlation values were over 0.7–0.8. Spearman’s rho value was used to grade the correlation between ratings of examiners as negligible (rho: 0.00–0.10), weak (rho: 0.10–0.39), moderate (rho: 0.40–0.69), strong (rho: 0.7–0.89), or very strong (rho: 0.90–1.00) [16]. We externally validated the HG system using an independent external cohort at Institution 2 and checked the inter-observer reliability for the HG system among examiners based on intra-class correlation and Spearman’s rho.

### 2.6. Perioperative Clinical Parameters

The primary endpoint was to determine correlations of clinical parameters with the severity of hematuria. Moreover, the basketing difficulty was evaluated as a secondary endpoint in RIRS. We recorded all patients’ clinical parameters that reflected surgical difficulties and between-group differences for each HG. These parameters included the Houns-field unit for stone density, stone number, stone size, total operative time, and Renal Stone Complexity score [17]. Difficulty during the basketing procedure was assessed using a modified visual analog system (range, 0–10; easiest experience = 0, most challenging experience = 10). In addition, we evaluated the correlation between the basketing difficulty of using a single-stone basket and the severity of hematuria after z-score transformation [(actual value − mean value)/SD] for normalization. Clinical data about the HG system and regarding one-month postoperative complications were obtained. Complications were categorized according to the HG system. Clinical stone-free status was defined as no evidence of any remnant stone > 2 mm.

## 3. Statistical Analysis

Patients grouped based on the HG system were compared using a one-way analysis of variance for continuous variables and a chi-square test for categorical variables. Categorical variables are presented with a number (percentage), and continuous variables with mean value ± standard deviation. Categorical and continuous variables are presented as percentages and mean values ± SD, respectively. Univariate and multivariate logistic regression analyses analyzed significant predictors of SFR and occurrence rates of acute pyelonephritis and sepsis. Statistical significance was considered at *p* < 0.05. All statistical analyses were performed with commercially available software such as IBM SPSS Statistics version 25 (IBM, Inc., Chicago, IL, USA) and R statistical software version 3.5.3 (www.r-project.org (accessed on 1 April 2022)).

## 4. Results

### 4.1. Patient Clinical Characteristics

Among about 3100 patients who underwent RIRS or mPCNL, 920 patients with relevant criteria who underwent RIRS (*n* = 669, 72.71%) and mPCNL (*n* = 251, 27.3%) during the study period were assessed. The mean age was 56.9 ± 14.1 years. Females accounted for 40.1%. Preoperative JJ stenting or percutaneous nephrostomy for RIRS and mPCNL were performed for 529 (57.5%) and 92 (10.0%) patients, respectively.

Table 2 shows the baseline characteristics of participants in each hematuria group. Development and external validation groups had 189 cases of RIRS and 142 cases of mPCNL. The HG system showed HG-zero in 21 (11.1%) and 28 (19.7%), HG-one in 92 (48.7%) and 63 (44.4%), HG-two in 59 (31.2%) and 27 (19.0%), HG-three in 17 (9.0%) and 23 (16.2%) patients, and HG-four in 0 (0%) and 1 (0.7%) patients in the RIRS and mPCNL groups, respectively. None of the patients met the HG four criteria in RIRS. No clinically significant between-group differences in clinical parameters were observed in either group. The distribution of cases and surgical outcomes of development and validation groups according to HG and surgical method are shown in Table 3.

### 4.2. Agreement among Examiners

In the development group, results showed high agreement for the HG system among examiners, with intra-class reliability of 0.928 [95% confidence interval, 0.899–0.950; *p* < 0.001] and 0.973 [95% confidence interval, 0.962–0.982; *p* < 0.001] for groups of RIRS and mPCNL patients, respectively. Strong correlations (Spearman’s correlation coefficient, range 0.846–0.903; *p* < 0.001, range 0.911–0.929; *p* < 0.001) were found for groups of RIRS and mPCNL patients, respectively. In the external validation group, a high agreement was shown for the HG system among examiners, with intra-class reliability of 0.964 [95% confidence interval, 0.948–0.975; *p* < 0.001] and 0.958 [95% confidence interval, 0.933–0.975; *p* < 0.001] for groups of RIRS and mPCNL patients, respectively. Strong correlations (Spearman’s correlation coefficient, range 0.876–0.918; *p* < 0.001, range 0.854–0.906; *p* < 0.001) were also found for groups of RIRS and mPCNL patients, respectively.

### 4.3. Correlations of Clinical Parameters with the Severity of Hematuria

Clinical parameters related to stones showed an increasing trend of surgical difficulty with increasing severity of hematuria (Table 3). The stone density of the Houns-field unit was the most important factor in determining the hematuria across the development and validation groups of RIRS and mPCNL. In the development group of RIRS, significant between-group differences in the Houns-field unit (*p* = 0.037), maximal diameter of stones (*p* = 0.027), stone volume (*p* = 0.005), operative time (*p* = 0.040), and the occurrence rates of acute pyelonephritis and bleeding without transfusion were observed according to the increase in the HG system. In the validation group of RIRS, only the Houns-field unit (*p* = 0.034) and the occurrence of acute pyelonephritis or sepsis (*p* < 0.001) showed significant between-group differences. In the development and validation groups of mPCNL, only the Houns-field unit showed significant between-group differences according to the increase in the HG system.

### 4.4. Prediction of Remnant Stones and the Occurrence of Inflammation with the Severity of Hematuria

Multivariate logistic regression analysis showed that the Houns-field unit (*p* = 0.040), the operative times (*p* = 0.001), and body weight (*p* = 0.022) were predictors for remnant stones in the RIRS group. However, the HG system (*p* = 0.009), preoperative creatinine (*p* = 0.012), Houns-field unit (*p* = 0.002), number of stones (*p* = 0.003), operative times (*p* < 0.001), and body weight (*p* = 0.010) were predictors for remnant stones in the PCNL group (Table 4 and Table 5).

Regarding the occurrence of acute pyelonephritis or sepsis, the HG system (*p* = 0.014), preoperative creatinine (*p* = 0.014), and the preoperative glomerular filtration rate (*p* = 0.023) were significant predictors in the RIRS group. However, preoperative creatinine (*p* = 0.010), the preoperative glomerular filtration rate (*p* = 0.022), and female gender were significant predictors in the mPCNL group.

### 4.5. Association of Stone Basketing Difficulty with HG in RIRS

**Figure 1:**
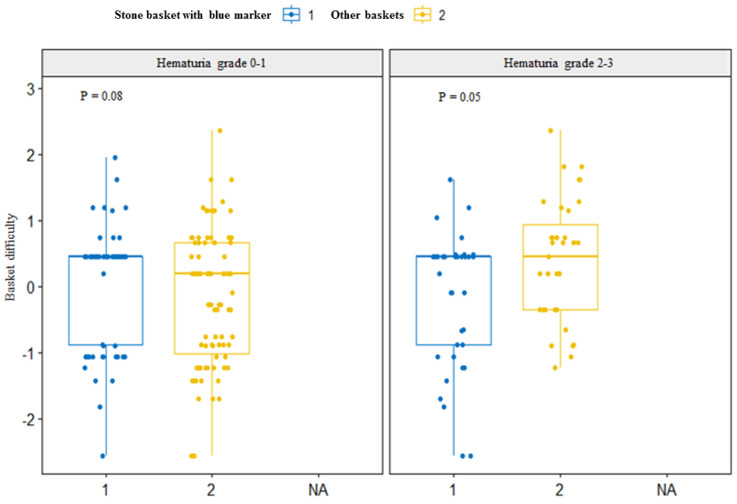
Assessment of basket visualization score for basketing difficulty after z-score normalization. Higher value represents more difficulty in basketing. Left side figure is for low hematuria grades of zero to one. Right side figure is for high hematuria grades of two to three. The blue box reflects stone basket with blue marker (group 1). The yellow box (group 2) reflects other baskets. Basket with blue marker shows better performance in high hematuria groups than in low hematuria groups.

Although no significant between-group differences in stone basketing difficulty score were observed (Figure 1), the average score notably increased in the HG-three group with blood clots (*p* = 0.21). Based on the severity of hematuria, we divided patients into low (HG-zero and HG-one) and high (HG-two, HG-three, and HG-four) hematuria groups. There was a significant difference in basketing difficulty between the low and high HG groups. In the high HG group, the surgical difficulty of basketing techniques was lower only in the basket with a blue marker instrument (Skylite) than in others, showing a borderline statistical significance (*p* = 0.05).

## 5. Discussion

In this study, we developed an easy and reliable HG system for RIRS and mPCNL that showed a strong agreement among examiners to assess hematuria. We found a significant association of the HGs with the pre- and intra-operative parameters, which can impact surgical outcomes. To the best of our knowledge, this is the first study to demonstrate the effectiveness of an HG system and the importance of blood clots for predicting surgical complications of RIRS and mPCNL.

Hematuria is one of the complications that leads to surgical difficulties and longer operative times. It affects the stone-free rate. Surgeons have tried to explain the level of surgical difficulty with intraoperative parameters. However, only the total operation time is a single candidate for explaining the level of surgical difficulty. Therefore, a validated HG system is warranted for clinicians to improve patient counseling and better evaluate surgical outcomes [18]. Quantifying the degree of hematuria severity is challenging due to several parameters, including the irrigated fluid velocity, degree of injury, operation field clarity affected by an endoscopic instrument, light source, irrigated fluid setting, and the surgical skill of a surgeon. Therefore, we employed simplified and easily applicable parameters in the surgical field that require no separate calculation. We developed a grading system based on the presence of a blood clot, any visible stone, and characteristic hematuria in every setting of irrigation fluid or instrument. This simplified approach might be beneficial for obtaining higher reliability among examiners with different endourological backgrounds. In our study, the HG system showed almost perfect intra-class reliability agreement and strong correlation among three certified urologists, four assistants, and nurses.

Previous studies showed that stone size, number, and location had been reported as risk factors for low stone-free rates [8,19,20]. The surgical outcome depended on the operation time, reflecting operation complications [20,21]. These studies did not include the parameters to show the surgical difficulty and the importance of blood clots. We found statistically significant associations between the severity of hematuria based on the HG system and perioperative parameters, reflecting the surgical difficulty in the present study.

In our study, the Houns-field unit, maximal diameter of stones, stone volume, operative time, and bleeding without transfusion were significantly associated with the increasing severity of hematuria. To treat hard and large-volume stones, a larger diameter of laser fiber, UAS, high-power laser settings, and other techniques are frequently used by surgeons. When we use high-power laser settings, tissue around the stone is more vulnerable to injury. That is why the above clinical parameters may cause severe hematuria. The high-power laser settings in our study are typically altered as follows. For RIRS, the initial laser power is usually 0.5 J, but it is usually increased to 1.0 or 1.2 J. For mPCNL, the initial laser power is usually 2.0 J, but it is sometimes increased to 2.5 J.

The high HG system group was a significant predictor for remnant stones in the PCNL group and the probability of acute pyelonephritis or sepsis in the RIRS group. The probability of acute pyelonephritis in the RIRS group can be increased by high-speed irrigation to clear the visual field. Thus, high HG system cases may lead to intraoperative high intrarenal pressure, which affects postoperative urinary tract infection [21,22]. In this situation, delicate control of the irrigation fluid is required for visibility in the high HG system group. A previous study showed that continuous flow irrigation at a fixed pressure might result in decreased pain, infection, and sepsis [23]. Further, the UAS was used to maintain lower intrarenal pressure [24]. However, when surgeons confront a closed renal collecting system accompanied by proximal ureteral narrowing, there is a limitation in reducing intrarenal pressure. Suction also can be one good solution to control the irrigation system, but rapid suction can also cause hematuria due to mucosa. Our HG system did not consider suction during RIRS; hence, the current HG system can be validated later again with the suction technique of RIRS.

Renal stone complexity based on stone number and location was significantly correlated with hematuria based on the HG system in our study. In addition, the present study showed that the high HG system of three and four with blood clots was another critical factor to consider in predicting the poor stone-free rates, because the blood clot can attach to the stone, and the surgeon can miss the stones inside the clots. Therefore, the surgeon needs to melt the clots or fragment the hidden stone fragments with a laser. We used long pulse laser emission to dissolve the clot, which reveals the chink in the clots and exposes the stone’s surface. In an additional way, surgeons can perform basketing techniques constantly after laser emission to see the maximal size of the stones. With these techniques, surgeons should be careful not to use high temperatures with continuous emission of a long-pulse laser for a long time. Therefore, with the above delicate surgical settings, our novel HG system successfully represented the importance of blood clots for high stone-free rates and the severity of operation difficulty with the HG system.

There was no significant between-group difference in basketing difficulty measured by a modified visual analog scoring system. However, a higher score was noted for the HG-three group. Interestingly, in the high hematuria subgroup with HG of three and four, the basket with a blue marker showed a trend for better performance than other instruments. In the usual setting of RIRS, stone basketing was performed by the first assistant. Although operators could use their hand–eye coordination for orientation, the first assistant measured the stone and basket distance through the direction of the eyes. In the high hematuria grade, complementary color contrast (green to blue) might aid in basket orientation, supporting better identification of structures already accepted for background sheet color during microsurgery [23]. In this study, we found evidence for optimal basketing selection in severe hematuria cases.

This study had several limitations. Firstly, there were no patients and one single patient with an HG-four grade score in the RIRS and mPCNL groups, respectively. This could be attributed to the stringent HG-four criteria, in which severe hematuria posed difficulty in proceeding with the operation. The rare occurrence of HG-four suggests RIRS and mPCNL as safe procedures in terms of bleeding. Therefore, we may consider removing HG-four from this grading system. However, we believe that surgeons might gain experience in managing HG-four levels of hematuria by handling such patients’ cases in rare situations. Secondly, this study was performed in a retrospective manner. Thus, it might not be free from selection bias. Lastly, the different types of flexible ureteroscope utilized may affect visibility. Different visibility may have an impact on HG evaluation. However, the HG system is based on the reaction of the surgeons regarding how to react with regard to the hematuria during surgery. Surgeons in this study thought that the type of ureteroscope might not be a detrimental factor in finding targeted stones in a bleeding situation [25].

Despite the shortcomings of this study, we successfully developed and validated the reliability of a novel HG system for RIRS and mPCNL. The new HG system showed significant associations with pre- and intra-operative parameters. Additionally, we found interesting evidence for optimal basket selection in severe hematuria conditions.

## 6. Conclusions

We developed a novel HG system for predicting surgical complications of minimally invasive stone surgery based on simplified and easily applicable parameters, including the presence of a blood clot, any visible stone, and characteristic hematuria in irrigation fluid. The new hematuria grading system shows excellent inter-observer reliability and a relationship with a gradual increase in surgical difficulty. The hematuria grade with clots was one of the significant predictors for remnant stones. The appropriate surgical technique is necessary to increase surgical outcomes with the condition of hematuria. In addition, the surgical difficulty of basketing techniques might be lower with the basket with a blue marker instrument than with others in the high hematuria group.

## Figures and Tables

**Table 1:** The newly developed grade system of hematuria for stone fragmentation during flexible ureteroscopy surgery and miniaturized percutaneous nephrolithotomy.

Grades	Description	Pictures
0	No hematuria with clear irrigation	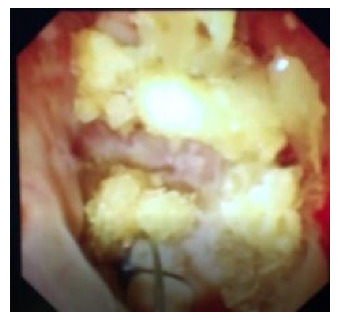
1	Mild hematuria with no clots, clearly visible stones	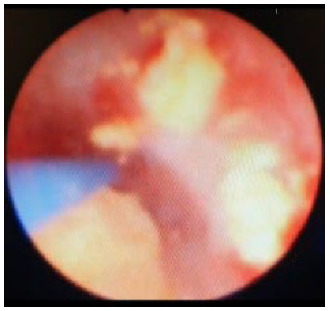
2	Scattering hematuria with no clots, visible stones	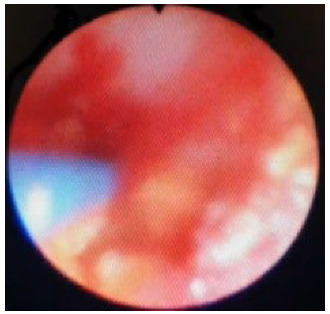
3	Hematuria with clots, no clearly visible stones	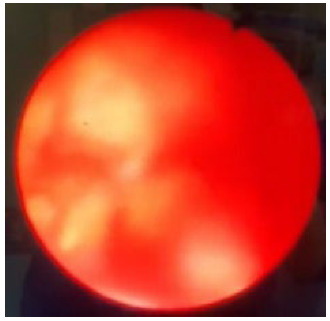
4	Unable or difficult to proceed with the surgery properly due to hematuria	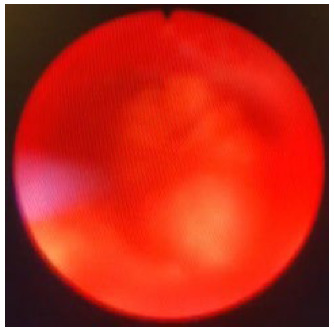

**Table 2:** Patient characteristics, clinical parameters, and complication rates of each grade group.

Hematuria Grade	0	1	2	3	4	*p*-Value
**RIRS group**						
Number of patients, n (%)	21 (11.1)	92 (48.7)	59 (31.2)	17 (9.0)	0 (0)	
Sex (Female), n (%)	8 (38.1)	42 (457)	23 (39.0)	3 (17.6)	N/A	0.206 ^‡^
Age (year)	59.0 ± 9.7	57.0 ± 13.5	53.6 ± 14.1	55.3 ± 9.3	N/A	0.384 ^†^
BMI (kg/m^2^)	25.6 ± 6.0	24.5 ± 3.8	24.3 ± 3.6	25.7 ± 4.3	N/A	0.497 ^†^
DM, n (%)	2 (25.0)	32 (30.5)	7 (11.9)	5 (29.4)	N/A	0.061 ^‡^
HTN, n (%)	2 (25.0)	55 (52.4)	23 (39.0)	9 (52.9)	N/A	0.209 ^‡^
Preoperative DJ insertion, n (%)	0 (0)	19 (18.1)	5 (8.5)	6 (35.3)	N/A	0.028 ^‡,^*
Preoperative acute, n (%) Pyelonephritis	0 (0)	12 (11.4)	4 (6.8)	1 (5.9)	N/A	0.551 ^‡^
Preoperative percutaneous nephrostomy insertion, n (%)	1 (12.5)	5 (4.8)	0 (0)	0 (0.0)	N/A	0.132 ^‡^
Preoperative serum creatinine (mg/dL)	1.3 ± 0.9	1.4 ± 1.4	1.1 ± 0.7	0.9 ± 0.2	N/A	0.181 ^†^
Preoperative GFR (ml/min/1.73 m^2^)	71.8 ± 30.8	77.0 ± 33.3	77.7 ± 28.0	82.5 ± 19.2	N/A	0.861 ^†^
Preoperative Hb (g/dL)	12.9 ± 0.9	13.2 ± 2.1	13.4 ± 2.3	13.7 ± 2.1	N/A	0.783 ^†^
**Miniaturized percutaneous nephrolithotomy group**						
Number of patients, n	28 (19.7)	63 (44.4)	27 (19.0)	23 (16.2)	1 (0.7)	
Sex (Female), n (%)	11 (39.3)	27 (42.9)	12 (44.4)	6 (26.1)	1 (100.0)	0.435 ^‡^
Age (year)	56.8 ± 11.0	59.4 ± 13.4	59.5 ± 12.7	61.9 ± 12.0	61.0	0.716 ^†^
BMI (kg/m^2^)	25.8 ± 2.9	24.7 ± 4.2	24.9 ± 3.0	25.5 ± 2.9	32.0	0.200 ^†^
DM, n (%)	5 (17.9)	12 (19.0)	5 (18.5)	3 (13.0)	1 (100.0)	0.296 ^‡^
HTN, n (%)	13 (46.4)	30 (47.6)	9 (33.3)	10 (43.5)	0 (0)	0.656 ^‡^
Preoperative DJ insertion, n (%)	20 (71.4)	42 (66.7)	11 (40.7)	15 (65.2)	0 (0)	0.073 ^‡^
Preoperative acute, n (%) Pyelonephritis	3 (10.7)	5 (7.9)	2 (7.4)	2 (8.7)	0 (0)	0.987 ^‡^
Preoperative percutaneous nephrostomy insertion, n (%)	4 (14.3)	13 (20.6)	10 (37.0)	5 (21.7)	0 (0)	0.551 ^‡^
Preoperative serum creatinine (mg/dL)	0.9 ± 0.3	1.0 ± 0.7	1.2 ± 1.1	0.9 ± 0.2	0.6	0.572 ^†^
Preoperative GFR (ml/min/1.73 m^2^)	87.1 ± 20.0	80.6 ± 26.7	78.5 ± 23.4	84.8 ± 16.3	98.2	0.581 ^†^
Preoperative Hb (g/dL)	13.1 ± 1.4	12.8 ± 1.8	13.3 ± 1.5	13.3 ± 1.3	12.3	0.513 ^†^

^†^: One-way ANOVA, ^‡^: Pearson chi-square, * *p* < 0.05.

**Table 3:** Surgical outcomes of the development and validation groups.

Hematuria Grade	0	1	2	3	4	*p*-Value
**RIRS in the development group (n)**	**8**	**65**	**23**	**4**	**0**	
Stone-free rates, n (%) ^‡^	7 (87.5)	60 (92.3)	19 (82.6)	2 (50.0)	N/A	0.064
Houns-field unit †	664.1 ± 538.8	831.0 ± 387.6	869.9 ± 385.2	1374.8 ± 224.8 *	N/A	0.037 *
No. of stones ^†^	2.5 ± 3.1	3.6 ± 6.8	4.6 ± 4.7	6.5 ± 6.9	N/A	0.659
Maximal diameter of stones ^†^	6.0 ± 3.6	7.2 ± 3.9	8.4 ± 3.6	12.3 ± 6.6 *	N/A	0.027 *
Volume of stones ^†^	285.0 ± 461.6	443.1 ± 797.0	540.4 ± 717.5	1998.3 ± 2231.1 *	N/A	0.005 *
Operative time (min) ^†^	50.9 ± 45.7	36.8 ± 22.9	40.0 ± 17.6	70.0 ± 31.6 *	N/A	0.040 *
Discharge, postoperative (day) ^†^	1.3 ± 0.5	1.2 ± 0.6	1.1 ± 0.5	1.3 ± 0.5	N/A	0.944
Acute pyelonephritis, n (%) ^‡^	2 (25.0)	0 (0)	1 (4.3)	2 (50.0)	N/A	<0.001 *
Bleeding without transfusion, n (%) ^‡^	0 (0)	0 (0)	0 (0)	1 (25.0)	N/A	<0.001 *
Bleeding with transfusion, n (%) ^‡^	0 (0)	0 (0)	0 (0)	0 (0)	N/A	N/A
Angioembolization, n (%) ^‡^	0 (0)	0 (0)	0 (0)	0 (0)	N/A	N/A
Mortality, n (%) ^‡^	0 (0)	0 (0)	0 (0)	0 (0)	N/A	N/A
Etc, n (%) ^‡^	0 (0)	0 (0)	0 (0)	0 (0)	N/A	N/A
**RIRS in the validation group (n)**	**13**	**25**	**36**	**13**	**0**	
Stone-free rates, n (%) ^‡^	12 (92.3)	25 (100.0)	31 (86.1)	9 (69.2)	N/A	0.207
Houns-field unit ^†^	600.7 ± 285.8	800.0 ± 408.3	845.9 ± 411.5	1078.0 ± 499.1 *	N/A	0.034 *
No. of stones ^†^	4.5 ± 4.3	2.5 ± 2.3	3.1 ± 3.0	2.8 ± 1.4	N/A	0.234
Maximal diameter of stones ^†^	19.0 ± 17.1	14.1 ± 15.1	13.3 ± 11.2	16.5 ± 10.7	N/A	0.570
Volume of stones ^†^	9200.6 ± 13,881.8	5041.6 ± 15,948.6	2933.6 ± 8388.1	2513.4 ± 3512.7	N/A	0.368
Operative time (min) ^†^	58.6 ± 35.5	46.3 ± 33.7	53.0 ± 37.3	80.0 ± 49.1	N/A	0.072
Discharge, postoperative (day) ^†^	1.3 ± 0.8	1.6 ± 1.2	1.4 ± 0.9	1.2 ± 0.4	N/A	0.608
Acuter pyelonephritis, n (%) ^‡^	0 (0)	0 (0)	0 (0)	3 (23.1)	N/A	<0.001 *
Bleeding without transfusion, n (%) ^‡^	0 (0)	0 (0)	0 (0)	0 (0)	N/A	N/A
Bleeding with transfusion, n (%) ^‡^	0 (0)	0 (0)	0 (0)	0 (0)	N/A	N/A
Angioembolization, n (%) ^‡^	0 (0)	0 (0)	0 (0)	0 (0)	N/A	N/A
Mortality, n (%) ^‡^	0 (0)	0 (0)	0 (0)	0 (0)	N/A	N/A
Etc., n (%) ^‡^	0 (0)	0 (0)	0 (0)	0 (0)	N/A	N/A
**Miniaturized percutaneous nephrolithotomy in the development group**	**25**	**38**	**13**	**15**	**1**	
Stone-free rates, n (%) ^‡^	22 (88.0)	32 (84.2)	9 (69.2)	10 (66.7)	1 (100.0)	0.099
Houns-field unit ^†^	958.4 ± 391.8	954.6 ± 394.0	1437.1 ± 506.3 *	1146.0 ± 353.0 *	525.0	0.003 *
No. of stones ^†^	3.5 ± 4.1	3.2 ± 3.7	3.0 ± 2.4	1.6 ± 2.3	2	0.538
Maximal diameter of stones ^†^	20.2 ± 9.3	29.5 ± 18.3	32.5 ± 15.9	26.8 ± 20.0	25.0	0.158
Volume of stones ^†^	4012.6 ± 4591.6	10,563.1 ± 20,470.0	21,742.1 ± 44,271.6	240,22.8 ± 79,180.7	6014.5	0.532
Operative time (min) ^†^	45.8 ± 26.3	73.8 ± 50.3	76.2 ± 38.7	58.2 ± 39.8	60.0	0.102
Discharge, postoperative (day) ^†^	1.6 ± 1.4	1.7 ± 1.4	1.7 ± 1.2	2.7 ± 2.6	4.0	0.173
Acuter pyelonephritis, n (%) ^‡^	1 (4.0)	6 (15.8)	3 (23.1)	1 (6.7)	1 (100.0)	0.393
Bleeding without transfusion, n (%) ^‡^	9 (36.0)	20 (52.6)	9 (69.2)	9 (60.0)	1 (100.0)	0.246
Bleeding with transfusion, n (%) ^‡^	1 (4.0)	4 (10.5)	1 (7.7)	5 (33.3)	1 (100.0)	0.076
Angioembolization, n(%) ^‡^	1 (4.0)	1 (2.6)	0 (0)	0 (0)	1 (100.0)	0.591
Mortality, n (%) ^‡^	0 (0)	0 (0)	0 (0)	0 (0)	0 (0)	N/A
Etc., n (%) ^‡^	2 (8.0)	0 (0)	0 (0)	0 (0)	0 (0)	0.256
**Miniaturized percutaneous nephrolithotomy in the validation group**	**3**	**25**	**14**	**8**	**0**	
Stone-free rates, n (%) ^‡^	3 (100.0)	19 (76.0)	13 (92.9)	7 (87.5)	N/A	0.448
Houns-field unit ^†^	1009.6 ± 251.9	1006.7 ± 310.3	1088.9 ± 420.3	1276.0 ± 474.1 *	N/A	0.034 *
No. of stones ^†^	6.3 ± 6.8	8.1 ± 25.1	11.8 ± 30.3	3.5 ± 3.3	N/A	0.891
Maximal diameter of stones ^†^	21.0 ± 8.2	24.1 ± 13.3	24.1 ± 7.9	26.4 ± 20.3	N/A	0.942
Volume of stones ^†^	1995.9 ± 1465.7	5465.4 ± 5818.5	4070.8 ± 3009.4	4507.5 ± 4019.2	N/A	0.614
Operative time (min) ^†^	50.0 ± 21.8	55.2 ± 34.4	69.9 ± 40.3	78.8 ± 36.9	N/A	0.321
Discharge, postoperative (day) ^†^	1.0 ± 1.0	2.5 ± 2.5	1.1 ± 0.5	1.3 ± 0.5	N/A	0.095
Acuter pyelonephritis, n (%) ^‡^	0 (0)	0 (0)	1 (7.1)	1 (12.5)	N/A	0.387
Bleeding without transfusion, n (%) ^‡^	0 (0)	4 (16.0)	2 (14.3)	1 (12.5)	N/A	0.899
Bleeding with transfusion, n (%) ^‡^	0 (0)	3 (12.0)	1 (7.1)	1 (12.5)	N/A	0.890
Angioembolization, n (%) ^‡^	0 (0)	0 (0)	0 (0)	2 (25.0)	N/A	N/A
Mortality, n (%) ^‡^	0 (0)	0 (0)	0 (0)	0 (0)	N/A	N/A
Etc., n (%) ^‡^	0 (0)	2 (8.0)	0 (0)	0 (0)	N/A	N/A

Categorical variables are presented with the number of patients (percentage) and continuous variables with mean value ± standard deviation. ^†^: One-way ANOVA, ^‡^: Pearson chi-square, * *p* < 0.05.

**Table 4:** Logistic regression analysis for prediction of remnant stones and the occurrence of acute pyelonephritis or sepsis in the development group of RIRS.

	Univariate		Multivariate	
	OR (95% CI)	*p*-Value	OR (95% CI)	*p*-Value
**Remnant stones**				
Hematuria grades (low versus high)	111.944 (0.125–100,620.0)	0.174		
Diabetes	0.001 (0.001–40.006)	0.176		
Hypertension	1.614 (0.059–40.340)	0.777		
Preoperative creatinine	2.338 (0.254–21.501)	0.453		
Preoperative glomerular filtration rate	1.051 (0.928–1.190)	0.431		
Houns-field unit	1.005 (1.001–1.011)	0.052	1.002 (1.001–1.005)	0.040 *
No. of stones	1.083 (0.944–1.244)	0.256		
Maximal diameter of stones	1.159 (1.075–1.248)	<0.001 *		
Operative times	1.165 (0.972–1.396)	0.099	1.104 (1.039–1.172)	0.001 *
Age	0.947 (0.849–1.056)	0.330		
Gender	1.635 (0.260–10.282)	0.600		
Height	0.891 (0.689–1.152)	0.378		
Body weight	0.917 (0.815–1.032)	0.149	0.885 (0.797–0.982)	0.022 *
**The occurrence of acute pyelonephritis or sepsis**				
Hematuria grades (low versus high)	1.457 (0.414–5.122)	0.558	2.152 (1.302–3.556)	0.014 *
Diabetes	0.673 (0.265–1.708)	0.405		
Hypertension	0.846 (0.421–1.699)	0.638		
Preoperative creatinine	1.284 (0.905–1.821)	0.161	1.678 (1.109–2.539)	0.014 *
Preoperative glomerular filtration rate	0.994 (0.979–1.010)	0.465	1.022 (1.003–1.042)	0.023 *
Houns-field unit	1.001 (1.010–1.025)	0.001 *		
No. of stones	1.124 (1.039–1.216)	0.004 *		
Maximal diameter of stones	1.025 (0.958–1.098)	0.475		
Operative times	1.018 (1.010–1.025)	0.001 *		
Age	0.977 (0.952–1.002)	0.073		
Gender	0.728 (0.086–6.179)	0.771		
Height	0.970 (0.918–1.025)	0.282		
Body weight	1.006 (0.964–1.050)	0.795		

Categorical variables are presented with the number of patients (percentage) and continuous variables with mean value ± standard deviation. * *p* < 0.05.

**Table 5:** Logistic regression analysis for prediction of remnant stones and the occurrence of acute pyelonephritis or sepsis in the development group of mPCNL.

	Univariate		Multivariate	
	OR (95% CI)	*p*-Value	OR (95% CI)	*p*-Value
**Remnant stones**				
Hematuria grades (low versus high)	7.299 (1.189–45.455)	0.010 *	1.622 (1.129–2.329)	0.009 *
Diabetes	0.673 (0.265–1.708)	0.405		
Hypertension	0.846 (0.421–1.699)	0.638		
Preoperative creatinine	1.284 (0.905–1.821)	0.161	1.383 (1.073–1.782)	0.012 *
Preoperative glomerular filtration rate	0.994 (0.979–1.010)	0.465		
Houns-field unit	1.000 (0.999–1.001)	0.619	1.002 (1.001–1.004)	0.002 *
No. of stones	1.124 (1.039–1.216)	0.004 *	1.126 (1.042–1.216)	0.003 *
Maximal diameter of stones	1.159 (1.075–1.248)	<0.001 *		
Volume of stones	1.000 (1.000–1.000)	0.009 *		
Operative times	1.018 (1.010–1.025)	0.001 *	1.017 (1.009–1.025)	<0.001 *
Age	0.977 (0.952–1.002)	0.073		
Gender	1.635 (0.260–10.282)	0.600		
Height	0.982 (0.944–1.022)	0.373		
Body weight	0.976 (0.946–1.022)	0.123	0.966 (0.941–0.992)	0.010 *
**The occurrence of acute pyelonephritis or sepsis**				
Hematuria grades (low versus high)	0.340 (0.045–2.555)	0.295	2.207 (1.341–3.632)	0.002 *
Diabetes	0.265 (0.053–1.317)	0.104		
Hypertension	1.634 (0.596–4.479)	0.340		
Preoperative creatinine	1.779 (1.153–2.746)	0.009 *	1.719 (1.138–2.598)	0.010 *
Preoperative glomerular filtration rate	1.024 (1.004–1.045)	0.020 *	1.023 (1.003–1.042)	0.022 *
Houns-field unit	0.999 (0.998–1.001)	0.355		
No. of stones	1.018 (0.927–1.118)	0.707		
Maximal diameter of stones	0.902 (0.924–1.024)	0.371		
Operative times	1.005 (0.992–1.019)	0.419		
Age	1.001 (0.962–1.039)	1.000		
Gender	2.960 (0.780–11.238)	0.111	2.286 (0.925–5.649)	0.073
Height	0.999 (0.924–1.080)	0.974		
Body weight	1.012 (0.968–1.060)	0.593		

Categorical variables are presented with the number of patients (percentage) and continuous variables with mean value ± standard deviation. * *p* < 0.05.

## Data Availability

The data presented in this study are available on request from the corresponding author.
